# Supplementary material for: Causal Relationship between Aging and Anorexia Nervosa: A White-Matter-Microstructure-Mediated Mendelian Randomization Analysis
Source: Biomedicines. 2024 Aug 16;12(8):1874. doi: 10.3390/biomedicines12081874 (PMC11351342; doi:10.3390/biomedicines12081874)
Supplement: Supplementary file 1 [file biomedicines-12-01874-s001.zip › Supplementary Figure/Figure S4/Singe SNP plot of MR analysis for TL to ubm-b-1620.pdf]

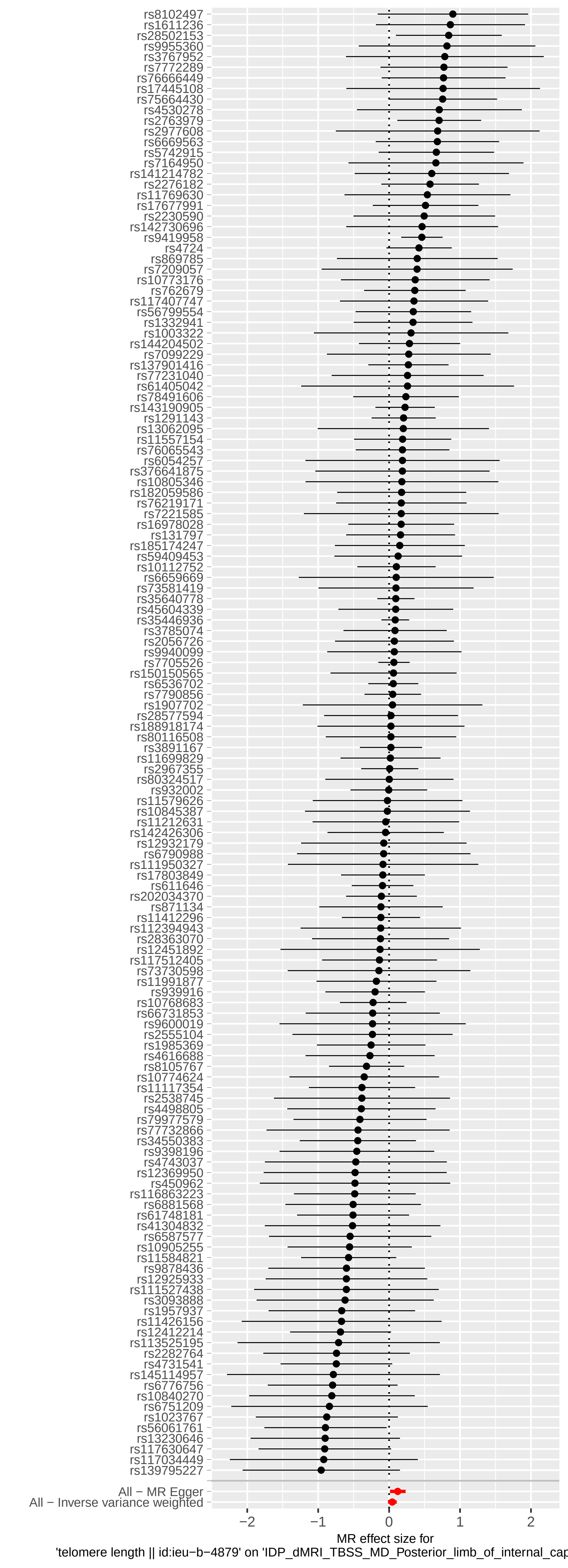
rs8102497
rs1611236
rs28502153
rs9955360
rs3767952
rs7772289
rs76666449
rs17445108
rs75664430
rs4530278
rs2763979
rs2977608
rs6669563
rs5742915
rs7164950
rs141214782
rs2276182
rs11769630
rs17677991
rs2230590
rs142730696
rs9419958
rs4724
rs869785
rs7209057
rs10773176
rs762679
rs117407747
rs56799554
rs1332941
rs1003322
rs144204502
rs7099229
rs137901416
rs77231040
rs61405042
rs78491606
rs143190905
rs1291143
rs13062095
rs11557154
rs76065543
rs6054257
rs376641875
rs10805346
rs182059586
rs76219171
rs7221585
rs16978028
rs131797
rs185174247
rs59409453
rs10112752
rs6659669
rs73581419
rs35640778
rs45604339
rs35446936
rs3785074
rs2056726
rs9940099
rs7705526
rs150150565
rs6536702
rs7790856
rs1907702
rs28577594
rs188918174
rs80116508
rs3891167
rs11699829
rs2967355
rs80324517
rs932002
rs11579626
rs10845387
rs11212631
rs142426306
rs12932179
rs6790988
rs111950327
rs17803849
rs611646
rs202034370
rs871134
rs11412296
rs112394943
rs28363070
rs12451892
rs117512405
rs73730598
rs11991877
rs939916
rs10768683
rs66731853
rs9600019
rs2555104
rs1985369
rs4616688
rs8105767
rs10774624
rs11117354
rs2538745
rs4498805
rs79977579
rs77732866
rs34550383
rs9398196
rs4743037
rs12369950
rs450962
rs116863223
rs6881568
rs61748181
rs41304832
rs6587577
rs10905255
rs11584821
rs9878436
rs12925933
rs111527438
rs3093888
rs1957937
rs11426156
rs12412214
rs113525195
rs2282764
rs4731541
rs145114957
rs6776756
rs10840270
rs6751209
rs1023767
rs56061761
rs13230646
rs117630647
rs117034449
rs139795227
All − MR Egger
All − Inverse variance weighted
−2
−1
0
1
2
MR effect size for
'telomere length || id:ieu−b−4879' on 'IDP_dMRI_TBSS_MD_Posterior_limb_of_internal_cap
